# A prospective randomized controlled multicentre trial comparing intravesical DMSO and chondroïtin sulphate 2% for painful bladder syndrome/interstitial cystitis

**DOI:** 10.1590/S1677-5538.IBJU.2016.0302

**Published:** 2017

**Authors:** Manuela Tutolo, Enrico Ammirati, Giulia Castagna, Katrien Klockaerts, Hendrik Plancke, Dieter Ost, Frank Van der Aa, Dirk De Ridder

**Affiliations:** 1 Department of Urology, University Hospitals, KU Leuven, Belgium;; 2 Division of Urology, Città della Salute e della Scienza, Molinette Hospital Hospital, University of Studies of Turin, Turin, Italy;; 3 Department of Urological Research Institute, IRCCS Ospedale San Raffaele, Division of Oncology/Unit of Urology, Milan, Italy;; 4 Department of Urology, St. Lucas Hospital, Gent, Belgium;; 5 Department of Urology, Imelda Hospital, Bonheiden, Belgium;; 6 Urology, St. Blasius Hospital, Dendermonde, Belgium

**Keywords:** Cystitis, Interstitial, Dimethyl Sulfoxide, Chondroitin Sulfates

## Abstract

**Objective:**

To compare effectiveness of intravesical chondroïtin sulphate (CS) 2% and dimethyl sulphoxide (DMSO) 50% in patients with painful bladder syndrome/interstitial cystitis (PBS/IC).

**Materials and methods:**

Patients were randomized to receive either 6 weekly instillations of CS 2% or 50% DMSO. Primary endpoint was difference in proportion of patients achieving score 6 (moderately improved) or 7 (markedly improved) in both groups using the Global Response Assessment (GRA) scale. Secondary parameters were mean 24-hours frequency and nocturia on a 3-day micturition dairy, changes from baseline in O’Leary-Sant questionnaire score and visual analog scale (VAS) for suprapubic pain.

**Results:**

Thirty-six patients were the intention to treat population (22 in CS and 14 in DMSO group). In DMSO group, 57% withdrew consent and only 6 concluded the trial. Major reasons were pain during and after instillation, intolerable garlic odor and lack of efficacy. In CS group, 27% withdrew consent. Compared with DMSO group, more patients in CS group (72.7% vs. 14%) reported moderate or marked improvement (P=0.002, 95% CI 0.05-0.72) and achieved a reduction in VAS scores (20% vs. 8.3%). CS group performed significantly better in pain reduction (-1.2 vs. -0.6) and nocturia (-2.4 vs. -0.7) and better in total O’Leary reduction (-9.8 vs. -7.2). CS was better tolerated. The trial was stopped due to high number of drop-outs with DMSO.

**Conclusions:**

Intravesical CS 2% is viable treatment for PBS/IC with minimal side effects. DMSO should be used with caution and with active monitoring of side effects. More randomized controlled studies on intravesical treatments are needed.

## INTRODUCTION

Because interstitial cystitis (IC) varies so much in symptoms and severity, most experts believe it is not one, but several diseases. In recent years, scientists have started to use the terms painful bladder syndrome (PBS) or bladder pain syndrome (BPS) to describe cases with painful urinary symptoms that may not meet the strictest definition of IC. IC can be considered as a subgroup of patients in whom cytoscopic findings can be noted. Painful bladder syndrome (PBS) is a chronic bladder condition characterized by chronic pelvic pain, pressure or discomfort perceived to be related to the bladder and accompanied by at least one urinary symptom, such as increased urinary urgency or frequency. The European Society for the Study Interstitial cystitis (ESSIC) decided to refer to the condition with the term “painful bladder syndrome/interstitial cystitis (PBS/IC)” (1). The American Urological Association (AUA) Guidelines Committee refers to an unpleasant sensation (pain, pressure, discomfort) perceived to be related to the urinary bladder, associated with lower urinary tract symptoms with a duration of at least 6 weeks, in the absence of any confusable diseases that may give rise to the symptoms (2, 3). The etiology of PBS/IC is still not well understood. To date, there is a general agreement on the administration of some oral or intravesical drugs (4, 5). With regard to intravesical therapy, it has been hypothesized that the urothelial mucin glycosaminoglycan (GAG) layer which protects the urothelial cells is damaged in PBS/IC (6, 7). Intravesical treatment with DMSO, chondroïtin sulphate, hyaluronic acid and heparin have been used to repair the GAG layer with variable clinical success (6-8). Dimethyl sulphoxide (DMSO, Rimso-50) is the only drug approved by the U.S. Food and Drug Administration (FDA) for intravesical treatment of PBS/IC. Only one small, short, single-center trial has reported efficacy (9). The EAU Guidelines on chronic pelvic pain updated in April 2014 underline that DMSO has been used in the past but there is insufficient current evidence to recommend its use (10). Chondroïtin sulphate (CS), an important component of the GAG layer seems to be promising, but comparative data with other therapies are lacking (11-13). A 2.0% solution of sodium CS in phosphate buffered saline (chondroïtin sulphate, Tribute Pharmaceuticals, Milton, ON) has been approved in Canada and Europe for intravesical treatment of PBS/IC. Assessing the outcome of such treatments is difficult. Objective parameters such as daytime and nighttime frequency may not always reflect the impact of the condition on the life of the patient. Patient reported outcome parameters are more frequently used to assess treatments in overactive bladder disease and in painful bladder research. Several validated questionnaires can be used to assess patients with PBS/IC. One of the most frequently used is the O’Leary-Sant questionnaire. Next to this questionnaire, the Global Response Assessment can be used. This is a validated 7 point Likert scale comparing the current status of the patient to the pre-intervention status. This scale has been used in several other studies on PBS/IC (11, 14-18). For the assessment of suprapubic pain the visual analog scale (VAS) was used. The aim of this study was to compare the clinical effectiveness of intravesical chondroïtin sulphate 2% (Uracyst™) and DMSO 50% in the treatment of patients with PBS/IC.

## MATERIALS AND METHODS

### Patients and study design

The study was designed as a prospective randomized multicenter evaluation of PBS/IC patients who were randomized to receive either 6 weekly instillations of CS 2% (treatment arm) or DMSO 50% (control arm). Four centers participated between January 2012 and January 2015, each center enrolling a similar number of patients. To detect a 0.75 difference on the 7 point Likert scale, with 80% power at 0.05% significance, 45 patients were needed in each group.

Candidates for inclusion in the study were men and women aged 18-75 years with a history of symptoms of bladder pain/discomfort described as suprapubic pain related to bladder filling, accompanied by other symptoms such as daytime and/or nighttime frequency in the absence of infection or other pathology. All patients underwent urodynamic evaluation and cystoscopy with an evidence of early bladder sensation and low maximum bladder capacity. We considered in this study also patients with negative macroscopic and bioptic findings of interstitial cystitis if a significant symptomatology was present. Patients should be willing and able to complete the necessary questionnaires.

The following patients were excluded from participating in this study: patients with transitional cell carcinoma of the bladder or other significant malignancy, pregnant or lactating, suffering from significant bacteriuria, diagnosis of hematuria, neurogenic bladder, indwelling catheters, chronic bacterial prostatitis, currently receiving or having received investigational drugs ≤30 days before screening, currently receiving or having had prior therapy with intravesical treatment (e.g. Uracyst, Cystistat®, heparin or Bacillus Calmette-Guérin (BCG)), receiving therapy for <3 months with antidepressants, anti-histaminics, hormonal agonists or antagonists; hence patient not stabilized on therapy (stable therapy defined as continuous treatment for ≥3 months), IC symptoms relieved by antimicrobials, anticholinergics or antispasmodics, functional bladder capacity of >400mL, neurologic disease affecting bladder function; any previous surgery or procedure having affected bladder function, current diagnosis of chemical, tuberculous or radiation cystitis, bladder or lower ureteral calculi, history of cancer within the last 5 years other than adequately treated non-melanoma skin cancers, active sexual transmitted disease, current vaginitis, endometriosis, any condition/disease which in the opinion of the investigator could interfere with patient compliance and/or interfere with the interpretation of the treatment results.

All patients gave written informed consent. Drop-outs and lost to follow-up are imputed as failures. Appropriate ethical approval was obtained according to national and international guidelines.

No compensation for the study was received by the patient, but the expense of the medical treatment and the medical consultations were free.

### Intervention

Patients were single-blind randomized to receive one intravesical instillation of 2.0% sterile solution of sodium chondroïtin sulfate (Uracyst™) or DMSO 50% once weekly for 6 weeks. Uracyst™ is delivered as a 2% sterile solution in 20mL vials. DMSO is prepared as a 50% solution in 50mL physiologic serum. The bladder instillation was performed by a trained medical professional. Using a temporary catheter, the bladder is filled with CS 2% or DMSO 50%. The patients were asked to retain the solution in their bladder for at least 15-30 minutes to allow it to work, and then urinate normally.

No antibiotic therapy was administered to the patient, but the catheterization technique was absolutely sterile. Eventual necessity of post-instillation antibiotic therapy was decided on personal decision of the doctor.

Our standard instillation schedule is: one instillation every week for 6 weeks, one instillation every month for 4 months, one instillation every 2 months for 6 months, one instillation every 3 months, if possible, for maintenance.

### Outcome assessment and study endpoints

Before and after treatment (at 7, 10 and 18 weeks), all patients were asked to fill in several questionnaires: the Interstitial Cystitis Symptom Index (4 questions) and Problem Index (4 questions) (ICSI/ICPI) (19) the Global Response Assessment (GRA) scale, the visual analog scale (VAS) for suprapubic pain, the 3-daily voiding diary, daily urinary frequency and nocturia. Each question in the O’Leary-Sant questionnaire was scored by the patient. Higher scores in each domain indicate greater symptom severity and impact on daily life. Maximum Symptom and Problem Index scores were 20 and 16, respectively (19). The GRA measures the overall improvement with therapy. The assessment asks: “As compared to when you started the current study (treatment), how would you rate your overall bladder symptoms now?”. The patient was provided with the following seven response options: markedly worse, moderately worse, slightly worse, no change, slightly improved, moderately improved and markedly improved. The VAS ranges from zero to 10 with zero representing no pain and 10 maximal pain. According to the study protocol the patients underwent 10 medical visits at weekly intervals for the first two months, thereafter at longer intervals. Table-1 presents the study overview.

**Table 1:** Study overview.

	Baseline	R/1	R/2	R/3	R/4	R/5	R/6	Q1	Q2	Q3

Week		1	2	3	4	5	6	7	10	18
VAS	x	x	x	x	x	x	x	x	x	x
Micturition diary	x							x	x	x
GRA								x	x	x
O'Leary-Sant	x							x	x	x
Instillation		x	x	x	x	x	x			
Adverse events		x	x	x	x	x	x	x	x	x

The primary outcome measure was the difference of the percentage of patients who achieved score 6 or 7 on the Global Response Assessment (GRA) scale comparing the patient’s present status with the pre-intervention status. A score of 6 indicates moderate improvement, while score 7 denotes marked improvement. Secondary parameters were the mean 24 hours frequency, nocturia episodes and functional bladder capacity measured on a 3-day micturition dairy, changes from baseline in the O’Leary-Sant questionnaire score and the assessment of suprapubic pain by the VAS.

Safety was assessed by monitoring adverse events at each visit. This publication shows a planned interim analysis for safety. A clinical evaluation committee evaluated the interim findings.

### Statistical analysis

An intention-to-treat analysis was performed. Statistical analysis of the patient questionnaire data was performed using Med Calc version 8.1 (Belgium) Statistical analyses were considered significant at a p-value less than 0.05.

## RESULTS

The analysis included 22 patients of both sexes (19 women and 3 men) treated with chondroïtin sulphate 2% and 14 (12 women and 2 men) treated with DMSO 50%. Baseline patient characteristics are presented in Table-2. The outcomes are shown in Table-3. For the primary outcome at 12 weeks, 72.7% of patients in the CS group achieved a GRA score of 6 or 7 compared with 14% of patients in the DMSO group (P=0.002, 95% CI 0.05-0.72). Decreases in pain (VAS), O’Leary-Sant nocturia and pain score compared to baseline were observed in both treatment groups and were statistically significant in the CS group. Although changes showed no statistically significant difference the CS group had a slightly smaller decrease in O’Leary-Sant IC total score (-7.2 points) compared to the DMSO group (-9.8 points). More than 50% of the patients in the DMSO group dropped-out (57%). The main reasons for treatment withdrawal in the DMSO group was the occurrence of side effects. Several patients reported pain during and after instillation, lack of efficacy, and intolerable garlic odor. Pain while holding DMSO in the bladder disappeared after voiding. By contrast, the drop-out rate in the CS group was only 27%. The main reasons were lack of efficacy or side effects. CS instillation side effects were all classified as Clavien-Dindo 1: urinary tract infection (n=2), urethral pain (n=2), dysuria (n=3).

**Table 2:** Baseline patient characteristics.

O’leary sant questionnaire score	All patients	Dmso group	Chondroitin group	
Urgency	3.9 ± 1.9	3.7 ± 2.0	3.8 ± 2.0	ns
Void within 2h	5.1 ± 1.5	5.0 ± 1.7	5.2 ± 1.4	ns
Nocturia	4.6 ± 1.5	4.7 ± 1.5	4.6 ± 1.4	ns
Pain and burning	4.7 ± 1.6	4.3 ± 1.7	4.7 ± 1.6	ns
VAS Pain	6.3 ± 2.3	6.2 ± 2.3	6.4 ± 2.3	ns
VAS urgency	7.5 ± 1.5	7.3 ± 1.5	7.7 ± 1.4	ns

**Total score**	**29.4 ± 10.8**	**29.8 ± 11.8**	**21.5 ± 16.0**	**ns**

**Table 3:** Drop-outs, Global Response Assessment, parameter changes vs. baseline.

	DMSO group	Chondroitin sulphate group
Drop-outs	8/14 (57%)	6/22 (27%)
GRA score 6 or 7	14.0%	72.7%
VAS reduction	8.3%	20%*
O'Leary total reduction	- 9.8 points	- 7.2 points
O'Leary nocturia subscale	4.7 to 4.0 (-0.7)	4.5 to 2.9 (-1.6)*
O'Leary pain subscale	4.3 to 3.7 (-0.6)	5.0 to 3.8 (-1.2)*

* statistically significant

Of the 16 patients who completed the CS treatment, all of them continued such treatment. We could not identify an ideal maintenance treatment schedule, as all patients received instillation cycles at different time intervals, according to the severity and recurrence of symptoms.

## DISCUSSION

Several intravesical drugs have been studied in the past, including heparin, lidocaine, pentosan polysulphate sodium, dimethyl sulfoxide (DMSO), chondroïtin sulphate (CS), hyaluronic acid (HA) (and combination with CS), as well as investigational drugs such as GM-0111. Recently, intravesical administration of botulinum toxin (BTX) has been studied in patients with PBS/IC (20).

A number of uncontrolled, open-label clinical studies have suggested that intravesical CS may have benefit in some PBS/IC patients with no significant safety issues (12, 21, 22). Steinhoff et al. (2003) showed beneficial effects of CS treatment in patients who have positive potassium stimulation test (PST) results (22). Daha et al. (2008) demonstrated that in patients who respond symptomatically to increased GAG substitution therapy, cystometric bladder capacity is increased, whereas non-responders experience a decrease in bladder capacity (7).

A previously published, prospective, but uncontrolled, multicenter, real-life clinical practice study suggested that intravesical CS 2% may have an important role in the treatment of IC. The study showed a response rate of 47% at 6 weeks, which increased with additional monthly treatment sessions to 60% at 24 weeks. In all, 48 of 53 patients (90.6%) had a positive PST. There were no significant safety issues during the study (11).

Two previously published, randomized, placebo controlled studies reported clinical benefit but failed to show statistically significant differences in improvement for 20mL weekly instillations of CS 2% after 6 and 8 weeks, respectively (16, 17).

Recently, individual participant data from an open-label study (OLS) (11) and 2 small randomized placebo controlled studies (RCTs) (16, 17) assessing intravesical CS 2% in PBS/IC were pooled (similar inclusion/exclusion criteria, treatment and outcome assessment). This meta-analysis including 213 patients confirms that CS does indeed provide significantly more benefit than placebo. At the end of treatment period (week 10 for OLS, week 7 for RCT1 and week 11 for RCT2), the overall GRA response rates were 43.2% (95% CI: 35.0, 51.5) and 27.4% (95% CI: 17.6, 37.2) for the CS and placebo groups, respectively. Pooled RR was 1.55 (P=0.014, 95% CI: 1.09, 2.22). The chance of having response to treatment was 55% significantly higher in the CS group than in the placebo group. The small decrease in total ICSI score and daily urine frequency between the two groups was less impressive (-0.8 and-0.5 points respectively) and not statistically significant. This underlines the importance of choosing the right patient for this treatment (23).

DMSO is approved in the U.S.A. as a standard therapy for intravesical treatment for PBS/IC. This is based on a small and old (1987) crossover study including 33 patients. Four intravesical treatments of 50mL 50% DMSO were administered at two-week intervals with 15 minutes retention. Patients were evaluated at one month post-treatment. When assessed subjectively, 53% of DMSO treated patients were markedly improved compared to 18% of the placebo treated patients. When assessed objectively (urodynamic assessment), 93% of the DMSO group and 35% of the placebo group were improved. No significant side effects to DMSO were noted (9). A further study conducted in 2000 by Peeker et al. demonstrated the superiority of instillations of DMSO over BCG in the reduction of pain and urinary frequency, but not of maximal functional capacity (24). The same studies are cited by a Cochraine review and by a NICE advice (25, 26). The recently AUA updated guidelines (2014) suggest limiting instillation dwell time to 15-20 minutes. DMSO is quickly absorbed into the bladder wall and longer periods of retaining are associated with significant pain. Side-effects include a garlic-like body odor in some people. This bothersome but relatively insignificant side effect may last up to 7 hours after treatment (27). Considering also a more recent study based on 28 patient that shows common side effects (48% of all the population) even using DMSO once a week for a 15-20 minute instillation (28), AUA guidelines suggest a prudent and controlled DMSO use (evidence-strength: grade C). In a recent work, Tomoe demonstrated that the population of patients with IC/BPS that mostly benefit from DMSO therapy is that with ulcerative Hunner lesions (29).

Our study is the first study in the literature comparing DMSO and CS for treatment of PBS/IC. The interim analysis of this study showed that CS 2.0% performed better than DMSO 50% in pain reduction and nocturia and in subjective outcome. CS 2.0% was also better tolerated than DMSO. Our data agree with those of a Downey et al. recent study, that found that intravesical CS reduced pain, urgency and O’Leary-Sant symptom and problem scores in patients with IC/PBS. All patients tolerated the treatment and no side effects were reported; a response to treatment was noted in patients who had failed a different intravesical bladder therapy (DMSO) (30). Both studies support the EAU guidelines recent update that do not recommend the standard DMSO use. We did not use a combined preparation of DMSO and lidocaine, to better identify the real direct benefit from DMSO without a possible confounding effect given by a simultaneous lidocaine instillation.

All patients had been adequately counselled and were aware about the possible incidence of side effects. Due to the high number of drop-outs in the DMSO arm caused by the high incidence of severe DMSO side effects, the clinical evaluation committee proposed to stop the study enrolment. Patients suffering of IC/BPS often complain of severe pain and urinary symptoms and come to medical attention to have effective and prompt treatments. It is not easy to achieve their full collaboration in a clinical trial and often do not accept the idea of a random assignment to a treatment option, requiring only the most effective. Patients were enrolled in this study during outpatient evaluation according to medical indication and patient desires. However, we can state that between 40% and 50% of patients declined enrollment because they wanted to choose the treatment and not accepting a random allocation.

Garlic odor is a typical feature of DMSO: patients were aware of this peculiar characteristic, but were blindly allocated to one of the two arms and never received confirmation by medical staff about the treatment they were receiving. Patient complaints were related more to side effects (pain and dysuria) than to the garlic odor.

Our results lead us to the conclusion that CS appears to be superior to DMSO in terms of efficacy and tolerability.

The limitations of this study are mainly the small sample size, the short follow-up and the lack of a placebo control group. On the other hand, randomization of patients to a placebo group may be difficult as PBS/IC patients were in severe pain and desperate for treatment. We did not do a washout period and then a crossover from one treatment to the other, this could represent a future implementation for this study.

To date, we have failed to stratify patients according to clinical phenotype because of the lack of proper biomarkers to categorize patients into groups that might respond differently to different interventions. A better approach for selecting patients with bladder-specific clinical phenotype might improve the overall response to intravesical CS 2% treatment (31).

## CONCLUSIONS

Intravesical chondroïtin sulphate 2% (Uracyst™) is a viable treatment for patients with PBS/IC with minimal side effects. DMSO, while being considered the gold standard should be used with caution and with active monitoring of side effects. Further large-scale prospective RCTs with long-term follow-up are needed to determine the long-term efficacy and durability of CS 2%.

## ARTICLE INFO

Int Braz J Urol. 2017; 43: 134-41
